# Application of stable‐isotope labelling techniques for the detection of active diazotrophs

**DOI:** 10.1111/1462-2920.13954

**Published:** 2017-12-15

**Authors:** Roey Angel, Christopher Panhölzl, Raphael Gabriel, Craig Herbold, Wolfgang Wanek, Andreas Richter, Stephanie A. Eichorst, Dagmar Woebken

**Affiliations:** ^1^ Division of Microbial Ecology, Department of Microbiology and Ecosystem Science Research network “Chemistry meets Microbiology,” University of Vienna Vienna 1090 Austria; ^2^ Division of Terrestrial Ecosystem Research, Department of Microbiology and Ecosystem Science Research network “Chemistry meets Microbiology,” University of Vienna Vienna 1090 Austria; ^3^ Present address: Biological Systems and Engineering Division, Lawrence Berkeley National Laboratory, Emeryville, CA, USA; Institute for Genetics, Technische Universität Braunschweig, Braunschweig, Germany

## Abstract

Investigating active participants in the fixation of dinitrogen gas is vital as N is often a limiting factor for primary production. Biological nitrogen fixation is performed by a diverse guild of bacteria and archaea (diazotrophs), which can be free‐living or symbionts. Free‐living diazotrophs are widely distributed in the environment, yet our knowledge about their identity and ecophysiology is still limited. A major challenge in investigating this guild is inferring activity from genetic data as this process is highly regulated. To address this challenge, we evaluated and improved several ^15^N‐based methods for detecting N_2_ fixation activity (with a focus on soil samples) and studying active diazotrophs. We compared the acetylene reduction assay and the ^15^N_2_ tracer method and demonstrated that the latter is more sensitive in samples with low activity. Additionally, tracing ^15^N into microbial RNA provides much higher sensitivity compared to bulk soil analysis. Active soil diazotrophs were identified with a ^15^N‐RNA‐SIP approach optimized for environmental samples and benchmarked to ^15^N‐DNA‐SIP. Lastly, we investigated the feasibility of using SIP‐Raman microspectroscopy for detecting ^15^N‐labelled cells. Taken together, these tools allow identifying and investigating active free‐living diazotrophs in a highly sensitive manner in diverse environments, from bulk to the single‐cell level.

## Introduction

The terrestrial nitrogen (N) cycle is essential for the Earth's biosphere and is intimately linked to microbial activity. Biological N_2_ fixation, that is, the reduction of atmospheric dinitrogen gas (N_2_) to ammonia, is of paramount importance as N is often a limiting factor for primary production. Excluding anthropogenic contributions, most of the fixed N in nature is provided through biological N_2_ fixation, which is performed by one guild of microorganisms (bacteria and archaea) – the N_2_ fixers or diazotrophs. Symbiotic (such as root nodule‐forming rhizobia) and nonsymbiotic (free‐living) diazotrophs contribute greatly to N_2_ fixation in terrestrial environments. Although the N_2_ fixation rate of symbiotic diazotrophs is higher than that of free‐living diazotrophs, the latter are of importance on a global scale due to their large numbers and wide range of distribution (Cleveland *et al*., 1999; Zehr *et al*., 2003; Gaby and Buckley, 2011) and possibly supply the majority of fixed N in environments such as deserts, temperate grasslands and tropical evergreen forests (Reed *et al*., 2011; Elbert *et al*., 2012). However, we still have a very limited understanding of these microorganisms in regard to their identity and the factors that govern there *in situ* activity primarily due to methodological challenges in studying them.

Historically, N_2_ fixation activity (or more specifically nitrogenase activity) has largely been evaluated at the process level through the use of the acetylene reduction assay (ARA) (Chalk *et al*., 2017). Although believed to be very sensitive, the theoretical estimates (for 1 molecule of N_2_, 3 molecules of C_2_H_2_ are reduced) can deviate significantly in practice (Nohrstedt, 1983). Furthermore, in environments such as soil, select microorganisms have the ability to use ethylene as a carbon source during these incubations (Zechmeister‐Boltenstern and Smith, 1998), thus, leading to an underestimation of the true acetylene reduction activity. Lastly, acetylene is known to be inhibitory or even toxic to certain groups of microorganisms, for example, methanotrophs (Bédard and Knowles, 1989), which are also known diazotrophs. The ^15^N tracer method is another well‐established method to measure N_2_ fixation. Samples are incubated with ^15^N_2_ gas and active diazotrophs assimilate ^15^N into their biomass (Montoya *et al*., 1996) and the accumulated isotopic tracer (isotopic enrichment) is a measure of activity. Traditionally, isotope measurements are performed on the entire sample (i.e., ‘bulk’ measurement) with a potential drawback of tracer dilution stemming from the large pool of passive N stored in soil organic matter, which could cause lowered sensitivity for detecting diazotrophic activity.

The genetic diversity and distribution of diazotrophs across many environments has been extensively investigated at the gene and transcript level by targeting the dinitrogenase reductase gene (*nifH*) (e.g. Riemann *et al*., 2010; Gaby and Buckley, 2011; Woebken *et al*., 2012; Collavino *et al*., 2014). However, since the process of N_2_ fixation is one of the most energy costly mechanisms in nature (requiring 16–24 mols of ATP for each mol of N_2_ fixed; Fisher and Newton, 2002) it is tightly regulated on multiple levels in the cell, such as the transcriptional‐ and post‐translational levels (Fischer, 1994; Dixon and Kahn, 2004). As a result, the presence of nitrogenase genes alone or even of their transcripts often does not reflect an accurate representation of the active diazotroph community and of their N_2_ fixation rates. To address this concern, the identity of active diazotrophs in a system can be revealed independent of using the marker gene by incubating the environmental sample with ^15^N_2_ in combination with density gradient centrifugation [stable isotope probing (SIP)] (Murrell and Whiteley, 2010). Thereby, ^15^N‐enriched DNA or RNA can be separated from unenriched DNA or RNA (^15^N‐DNA‐SIP and ^15^N‐RNA‐SIP, respectively), which allows an investigator to target microorganisms actively incorporating the ^15^N into biomass. When combined with high‐throughput sequencing, this powerful tool provides a window into the identity of the active participants in the N_2_ fixation process (Buckley *et al*., 2007a, 2007b). In contrast to ^15^N‐DNA‐SIP, the feasibility of ^15^N‐RNA‐SIP has only been shown with an enrichment culture (Addison *et al*., 2010), and has not yet been successfully demonstrated in environmental samples. RNA‐SIP has multiple advantages over DNA‐SIP, such as rapid tracer incorporation, labelling which is independent of cellular replication (Lueders *et al*., 2016) and no suffering from the ‘G + C effect’. As such, optimization of existing ^15^N‐SIP tools, especially with a focus on ^15^N‐RNA‐SIP, is warranted for studying active diazotrophs in highly diverse systems such as terrestrial samples.

Raman microspectroscopy is a rapid, vibrational spectroscopic method (Huang *et al*., 2004; Jarvis and Goodacre, 2004), allowing an investigator to track the incorporation of a stable isotope [such as ^13^C or deuterium (D)] into single cells (Huang *et al*., 2007; Berry *et al*., 2015). Since it is a nondestructive method, it can be coupled with downstream cultivation or molecular analyses. It permits an investigator to link physiological function to uncultivated microorganisms as illustrated with naphthalene degradation (Huang *et al*., 2007), phenylalanine uptake (Haider *et al*., 2010), carbon dioxide fixation (Li *et al*., 2012) or as a general activity marker when combined with D_2_O (Berry *et al*., 2015). Raman microspectroscopy has recently been applied in combination with D_2_O incubations in soil systems to detect active single cells (Eichorst *et al*., 2015), but has primarily been used to classify soils and to characterize soil structure and/or mineral content (e.g., Corrado *et al*., 2008; Luna *et al*., 2014). While the detection of ^15^N‐enriched cells and quantification of ^15^N‐labelling via Raman microspectroscopy has been shown for *E. coli* (Wang *et al*., 2013; Muhamadali *et al*., 2015; Wang *et al*., 2016), to the best of our knowledge, there has been no comprehensive investigation of its potential use for other strains or for environmental samples.

In this study, we evaluated the sensitivity of detecting diazotrophic activity in soils using the ARA and the ^15^N_2_ tracer method. We further assessed the sensitivity of detecting diazotrophic activity by tracing ^15^N incorporation into different biomolecules extracted from ^15^N_2_‐incubated soil samples in comparison to the traditional method of whole‐sample or ‘bulk’ analysis. We optimized and further developed a ^15^N‐RNA‐SIP approach for environmental samples, and demonstrated the applicability of this approach for the detection of active diazotrophs in a beech forest soil and benchmarked these results to ^15^N‐DNA‐SIP. Lastly, we investigated the feasibility of using ^15^N‐Raman microspectroscopy to detect ^15^N‐labelled cells across a collection of phylogenetically diverse strains with varying degrees of ^15^N enrichment.

## Results and discussion

### Comparison of ARA and ^15^N tracer assays

**Figure 1:**
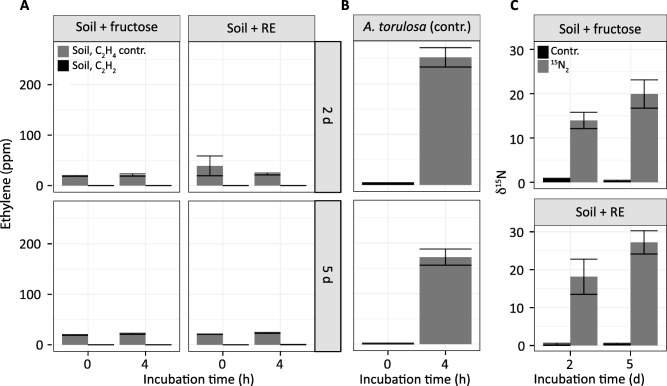
Comparison of ARA and ^15^N_2_ tracer assay. ARA performed on a forest soil sample incubated with either fructose or artificial root exudates (RE) (panels A) and a cyanobacterial culture (*A. torulosa*) (panels B). Legend for panels A and B is located in panel A. ‘Soil, C_2_H_4_ contr.’ indicates an ethylene consumption control performed on the forest soil sample as described in Experimental procedure. ‘Soil, C_2_H_2_’ refers to soil samples incubated with acetylene in the ARA. Graphs depict average ethylene concentration (ppm) ± standard error. Panels C depict the incorporation of ^15^N from ^15^N_2_ gas (average δ^15^N ± standard error) in the same soil samples used for ARA. ‘Contr’. indicates incubation with lab air.

ARA and ^15^N_2_ tracer assays are both classical and well‐established techniques for quantifying N_2_ fixation in environmental samples. While ^15^N_2_‐tracer assays are commonly used in marine science (Marchant *et al*., 2016), its application in terrestrial systems lags behind that of the ARA (Chalk *et al*., 2017). Here, we assessed the applicability of the ^15^N_2_‐tracer assay on low‐activity soil samples in comparison to the ARA to determine the optimal (more sensitive) method for quantification of diazotrophic activity. For this comparison, ^15^N_2_ tracer assays and ARAs were performed on the same batch of samples, the latter after 2 or 5 days of incubation with ^15^N_2_. Ethylene was not detected in soil samples supplemented with fructose or artificial root exudate (Fig. 1A), yet about 200 ppm ethylene was detected in the control (liquid culture of *Anabaena torulosa;* Fig. 1B). Ethylene concentrations in controls remained constant throughout the incubation; hence illustrating that ethylene was not consumed (Fig. 1A). In contrast, N_2_ fixation activity was detected in these samples by analysis of ^15^N incorporation in bulk soil samples using IRMS (^15^N_2_‐tracer assay) showing enrichments ranging on average from 14‰ to 18‰ (day 2) and 20‰ to 27‰ (day 5) compared to natural abundance controls (0.5‰ on average) (Fig. 1C).

As soil ethylene consumption did not interfere with the ARA, we hypothesized that the nitrogenase activity in these samples was below the quantification limit of this assay. To test this hypothesis, the amount of ethylene produced based on the measured ^15^N enrichment was estimated and compared with the quantification limit of the ARA in our laboratory. Based on the theoretical 3:1 conversion factor of acetylene relative to N_2_ by nitrogenase (Nohrstedt, 1983), we calculated that about 1.4 to 3.8 ppmv of ethylene was produced during the 4 h incubation, well below the quantification limit of about 5 ppmv ethylene by our GC method.

While the ARA is often the method of choice for quantifying N_2_ fixation potential in environmental samples, we were unable to detect any nitrogenase activity in this forest soil using the ARA and illustrated that the ^15^N_2_‐tracer assay was more sensitive. However, this may depend on the amount of nitrogen already present in the system. Similar results, showing that ^15^N_2_‐tracer assays can be more sensitive than ARA, have been obtained for oligotrophic marine samples (Montoya *et al*., 1996). Taken together, our results demonstrate that measuring the isotopic incorporation (^15^N_2_ tracer assay) can be more sensitive than the ARA, highlighting the difficulty in applying ARA to environments where diazotrophic activity is expected to be low or sporadic, such as in soils.

### Tracing ^15^N incorporation into biomolecules

The use of the ^15^N_2_‐tracer assay, by measuring ‘bulk’ ^15^N enrichment, to detect and quantify the activity of diazotrophs in the environment is well established and has been widely applied (Marchant *et al*., 2016; Chalk *et al*., 2017). Yet, the ‘bulk’ ^15^N measurement is not only dependent on the activity of the diazotrophs but also on the size of the background N pool (primarily stemming from soil organic matter), which can lead to isotopic dilution resulting in lower sensitivity in detecting N_2_ fixation. As such, we hypothesized that by targeting specific microbial biomolecules, such as nucleic acids or proteins, one could attain a higher level of sensitivity with the ^15^N_2_‐tracer assays as the N background of soil organic matter would not influence the isotopic enrichment of such biomolecules.

**Figure 2:**
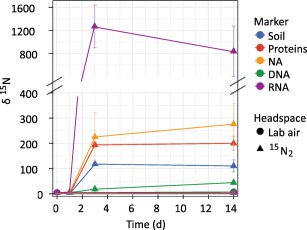
Average ± standard error of ^15^N enrichment in bulk soil and different microbial biomolecules measured by EA‐IRMS. Forest soil samples were incubated for up to 14 days in an atmosphere containing ^15^N_2_, or in lab air as a control.

Enrichment in ^15^N was detected in DNA, RNA, proteins and bulk soils after 3 days of incubation (Fig. 2). The highest ^15^N enrichment was detected in RNA (δ^15^N 1264 ± 370‰, mean ± SEM), followed by nucleic acids (NA, 224 ± 96‰), proteins (194 ± 9‰,), the bulk soil (118 ± 6‰) and DNA (5 ± 3‰) after 3 days. More specifically, δ^15^N values from RNA were up to ten times higher than those found in bulk soil or other biomolecules. Typically, ^15^N enrichment was the highest after 3 days, except for DNA where values rose to 16 ± 6‰ at day 14. The level of ^15^N‐enrichment in DNA was lower even than that in the bulk soil, indicating little growth of diazotrophs despite being active and synthesizing new RNA and proteins. Alternatively, it could reflect a large background of DNA compared with RNA in dead cells or in form of extracellular DNA within the soil (Carini *et al*., 2016). Values of δ^15^N for RNA decreased to 835 ± 437‰ at day 14 (Fig. 2), suggesting a decrease in nitrogenase activity and potential use of other N sources. This experiment demonstrates that by targeting microbial macromolecules, namely RNA and proteins, one is able to detect ^15^N incorporation after shorter incubation times and with greater sensitivity compared to traditional bulk soil measurements.

### Evaluating the ^15^N‐RNA‐ and DNA‐SIP approach for detecting soil diazotrophs

#### Applying ^15^N‐RNA‐SIP to soil samples

Given that the ^15^N enrichment of RNA was higher than that of DNA, we believed that ^15^N‐SIP investigations targeting the RNA pool as compared to the DNA pool could give a more sensitive and, thus, representative picture of the active diazotrophic community. As a first step, we compared ^15^N‐RNA‐SIP to ^15^N‐DNA‐SIP in pure cultures with varying G + C content. A mixed RNA sample containing fully ^15^N‐labelled RNA of a low G + C bacterium (*Flavobacterium johnsoniae*, genomic G + C content = 34%) and unlabelled RNA from a high G + C bacterium (*Pseudomonas putida*, genomic G + C content = 62%) was successfully separated using a CsTFA density gradient (Supporting Information and Fig. S1). However, separating the mixed DNA of these cultures in a CsCl gradient was unsuccessful, presumably due to the G + C effect as previously reported (Buckley *et al*., 2007a; Supporting Information and Fig. S1).

**Figure 3:**
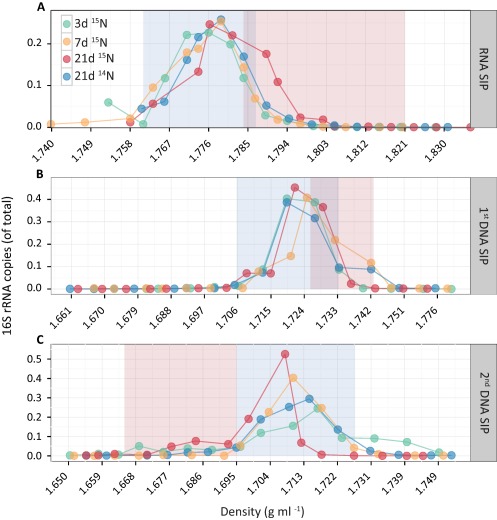
Proportion of bacterial 16S rRNA copies recovered from each of the SIP gradient fractions in ^15^N‐RNA or ‐DNA SIP experiments of soil samples: RNA‐SIP (CsTFA density gradients; panel A); primary DNA‐SIP (CsCl density gradients; panel B); and secondary DNA‐SIP (CsCl density gradients with bis‐benzimide; panel C). Values on the *Y*‐axis represent the proportion of the 16S rRNA copies out of the total number of copies of the entire gradient. Blue‐ and red‐shaded areas indicate the fractions where unlabelled and labelled template is expected to concentrate, respectively. The legend for all panels is located in panel A. “^15^N” in the legend refers to samples incubated in artificial atmosphere containing ^15^N_2_ gas, “^14^N” refers to control incubations in lab air.

In contrast to the results from pure cultures, no peak separation was observed after density gradient centrifugation of RNA originating from soil samples, which were incubated in the presence of ^15^N_2_ gas for 3, 7 or 21 days (Fig. 3A). Similarly, when analysing DNA from the same soil samples, we also observed no peak separation after the primary or after the secondary centrifugation step with bis‐benzimide (Fig. 3B and C). We believe the lack of peak separation for the soil samples was due to the low amount of isotopically ‘heavy’ template migrating to the part of the gradient where ^15^N‐labelled templates are expected to concentrate. Peak separation is challenging to achieve for ^15^N‐labelled nucleic acids since even fully labelled RNA will only be about 0.015 g ml^−1^ denser than unlabelled RNA (compared to about 0.035 g ml^−1^ difference in the case of carbon or oxygen isotope enrichment (Lueders *et al*., 2003; Angel and Conrad, 2013). As such, ^15^N‐labelled RNA will migrate to regions of the gradient where the background from unlabelled RNA is still relatively high, thus causing a lack of peak separation. Furthermore, since active free‐living diazotrophs are expected to constitute only a minor fraction of the total bacteria (Levy‐Booth and Winder, 2010; Orr *et al*., 2011; Harter *et al*., 2014), even if some diazotrophs have fully incorporated the ^15^N label they might not contribute sufficient RNA to the ‘labelled’ fractions in order to visibly surpass the background level.

**Figure 4:**
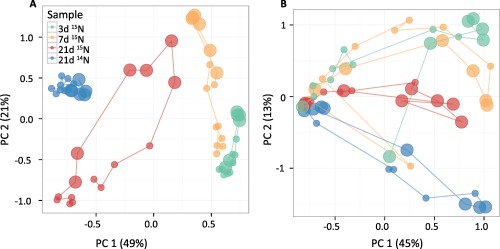
Principal coordinates analysis (PCoA) depicting the Morisita–Horn dissimilarities among microbial communities in the different SIP fractions from RNA (panel A) and the secondary DNA gradient (panel B). Samples from each SIP gradient are connected by a line to aid interpretation. Large circles depict fractions where the majority of ^15^N‐enriched RNA (ranging from 1.785 to 1.820 g mL^−1^) and DNA (ranging from 1.665 to 1.695 g mL^−1^) were expected to be found. Legend for both panels can be found in panel A.

To test this theory, we sequenced each of the collected fractions from the RNA‐SIP and the secondary DNA‐SIP gradients using universal SSU rRNA primers and performed a beta‐diversity analysis of the communities. Beta‐diversity analysis of the microbial communities depicted a clear shift in the community of the fractions where labelled RNA is expected in the RNA gradients at day 7 and 21, along the second axis (Fig. 4A). A minor shift is also seen at day 3, but it does not seem to be greater than the pattern seen for the control gradient (21 days, unlabelled). In contrast, no clear pattern of separation of fractions could be observed in the secondary DNA SIP gradients (Fig. 4B). While a separation of samples (SIP fractions) can be clearly seen along the first axis of the ordination, there does not seem to be a clear difference between the control and the labelled samples. The observed separation patterns in the DNA‐SIP could be due to differences in G + C content, or simply reflect sequencing noise.

#### Identifying specific OTUs involved in ^15^N_2_ fixation using RNA‐SIP

The microbial community across all fractions stemming from the RNA‐ and DNA‐SIP gradients (and controls) were characterized using high‐throughput sequencing targeting the 16S rRNA and the 16S rRNA gene, respectively, in an attempt to detect the labelling of minor members of the microbial community as described previously (Zemb *et al*., 2012; Aoyagi *et al*., 2015; Pepe‐Ranney *et al*., 2016). Sequencing of 120 fractions from RNA‐SIP and DNA‐SIP gradients produced a total of 561 195 paired reads, of which 367 425 (65%) were retained after contig assembly, quality filtering and chimera removal: 203 489 were assigned to RNA‐SIP fractions, while 163 936 were assigned to DNA‐SIP fraction. The sequences were clustered into 2668 OTUs based on 97% identity: 2469 assigned to RNA‐SIP fractions and 2410 assigned to DNA‐SIP fractions. Approximately 83% of the OTUs were shared between the RNA‐SIP and DNA‐SIP fractions. Each sample (SIP fraction) on average was assigned 3062 ± 158 reads, clustered into 494 ± 19 OTUs. After filtering sparse OTUs, 328 OTUs remained in the RNA‐SIP fractions and 335 in the DNA‐SIP fractions.

**Figure 5:**
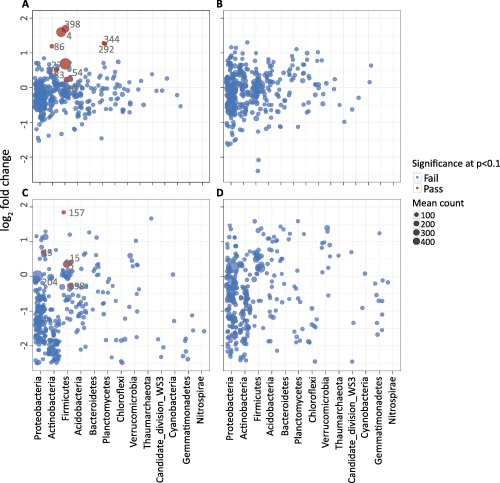
Fold change (log_2_) of OTUs between labelled and unlabelled fractions in each SIP‐gradient: RNA‐SIP gradients from ^15^N_2_ incubated samples (panel A); RNA‐SIP gradients from ^14^N_2_ incubated samples (panel B); DNA‐SIP gradients from ^15^N_2_ incubated samples (panel C); DNA‐SIP gradients from ^14^N_2_ incubated samples (panel D). Each dot represents an OTU, which passed sparsity filtering (see Experimental procedure). The *X*‐axis shows the mean normalised counts of the OTUs across all samples for each displayed phylum, while the *Y*‐axis is the mean log_2_ fold change across all gradients. Red dots denote OTUs enriched in the labelled fractions compared to the control, with log_2_ fold change of >0.25 and an adjusted *P*‐value of <0.1.

Eleven OTUs were significantly enriched in the ‘labelled’ fractions of the RNA‐SIP gradients and six OTUs were significantly enriched in the ‘labelled’ fractions of the DNA‐SIP gradients (Fig. 5A and C respectively; highlighted in red) after modelling the data using DESeq2. Not only did we detect more OTUs with RNA‐SIP, but also a broader phylogenetic distribution of the OTUs encompassing members of the *Proteobacteria*, *Actinobacteria*, *Firmicutes* and *Planctomycetes* (Table 1). A similar analysis of the control gradients (unlabelled RNA‐ and DNA‐SIP gradients) did not yield any significant OTUs (Fig. 5B and D), providing confidence in the method and the model parameters. In addition, the OTUs detected as labelled by the model exhibited higher log_2_ fold changes in the RNA‐SIP compared to DNA‐SIP (Fig. 5A and C).

**Table 1:** Taxonomic classification of the enriched OTUs in the RNA‐ and DNA‐SIP gradients.

RNA‐SIP	Domain	Phylum	Class	Order	Family	Genus
**OTU 2** a	**Bacteria**	**Firmicutes**	**Clostridia**	**Clostridiales**	**Clostridiaceae**	**Clostridium_sensu_stricto**
**OTU 4**	**Bacteria**	**Firmicutes**	**Clostridia**	**Clostridiales**	**Clostridiaceae**	**Clostridium_sensu_stricto**
**OTU 398**	**Bacteria**	**Firmicutes**	**Clostridia**	**Clostridiales**	**Clostridiaceae**	**Clostridium_sensu_stricto**
**OTU 20**	**Bacteria**	**Firmicutes**	**Bacilli**	**Bacillales**	**Bacillaceae**	**Bacillus**
OTU 83	Bacteria	Actinobacteria	Actinobacteria	Streptomycetales	Streptomycetaceae	Streptomyces
OTU 27	Bacteria	Actinobacteria	Actinobacteria	Streptomycetales	Streptomycetaceae	Streptomyces
OTU 54	Bacteria	Firmicutes	Bacilli	Bacillales	Paenibacillaceae	Paenibacillus
OTU 272	Bacteria	Proteobacteria	Alphaproteobacteria	Rhizobiales	Bradyrhizobiaceae	Rhodopseudomonas
OTU 86	Bacteria	Actinobacteria	Actinobacteria	Micrococcales	Micrococcaceae	Arthrobacter
OTU 292	Bacteria	Planctomycetes	Planctomycetacia	Planctomycetales	Planctomycetaceae	Unclassified
OTU 344	Bacteria	Planctomycetes	Planctomycetacia	Planctomycetales	Planctomycetaceae	Unclassified
**DNA‐SIP**	**Domain**	**Phylum**	**Class**	**Order**	**Family**	**Genus**
**OTU 2**	**Bacteria**	**Firmicutes**	**Clostridia**	**Clostridiales**	**Clostridiaceae**	**Clostridium_sensu_stricto**
**OTU 398**	**Bacteria**	**Firmicutes**	**Clostridia**	**Clostridiales**	**Clostridiaceae**	**Clostridium_sensu_stricto**
OTU 15	Bacteria	Firmicutes	Clostridia	Clostridiales	Clostridiaceae	Clostridium_sensu_stricto
OTU 43	Bacteria	Proteobacteria	Gammaproteobacteria	Alteromonadales	Idiomarinaceae	Aliidiomarina
OTU 157	Bacteria	Firmicutes	Clostridia	Clostridiales	Clostridiaceae	Clostridium_sensu_stricto
OTU 204	Bacteria	Proteobacteria	Alphaproteobacteria	Caulobacterales	Caulobacteraceae	Brevundimonas

a Rows in bold represent highly enriched OTUs.

**Figure 6:**
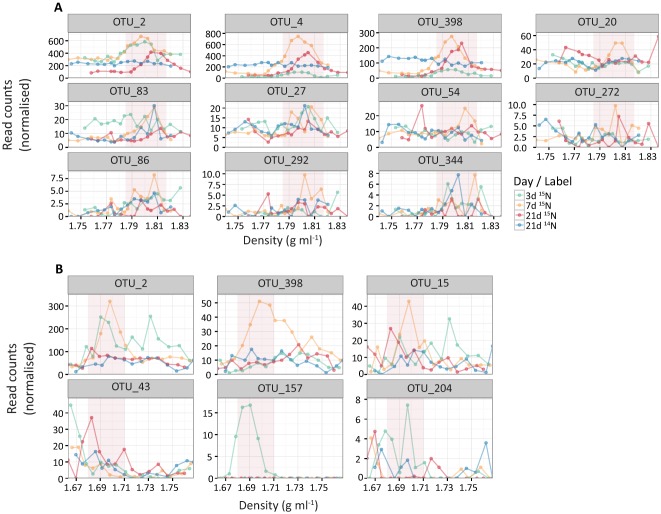
Normalised OTU abundances of enriched OTUs along the density gradients from RNA‐SIP (panel A) and DNA‐SIP (panel B). Shaded areas indicate where labelled template is expected to concentrate.

Although RNA‐SIP appears to detect more ^15^N‐enriched phylogenetically diverse OTUs as compared to DNA‐SIP (11 vs. 6), the normalized read count for 7 of the OTUs were less than 1.25, as compared to isotopically enriched OTUs identified by DNA‐SIP (only 1 OTU had read counts <8; Fig. 6). These OTUs comprised on average only 0.05–0.46% of the reads. Plotting their abundance distribution along the gradient fractions revealed a typical ‘hump‐shaped’ distribution for OTUs 2, 4, 398 and 20 in the RNA‐SIP gradients (Fig. 6A). OTU 2 appears to have been strongly labelled already at day 3, while OTUs 4 and 398 were only weakly labelled at day 3 but strongly labelled at days 7 and 21. OTUs 20, 83 and 292 displayed a ‘hump‐shaped’ distribution only in the gradients of day 7, while OTUs 27, 54, 272, 86 and 344 did not display a ‘hump‐shaped’ distribution at all, despite having a higher abundance in some ‘labelled’ fractions compared to the ‘unlabelled’ fractions. Across the DNA‐SIP gradients, a ‘hump‐shaped’ distribution was observed for OTU 2 (at day 3 and 7), OTU 398 (at day 7) and OTU 157 (at day 3). OTUs 15, 43 and 204 appeared to be ^15^N‐enriched at day 7, 21 and 3, respectively, but did not display a ‘hump‐shaped’ distribution (Fig. 6B).

Based on this visual inspection and considering the abundances of the OTUs that were detected by the model, we consider OTUs 2, 4, 398 and 20 as truly isotopically enriched in the RNA‐SIP and OTUs 2 and 398 in the DNA‐SIP experiments. These OTUs were members of the phylum *Firmicutes*, namely members of the families *Clostridiaceae* (OTUs 2, 4 and 398) and *Bacillaceae* (OTU 20; Table 1). Although members of the phylum *Firmicutes* are common to temperate forest soils (Baldrian *et al*., 2012; Hartmann *et al*., 2014), it was interesting to observe OTUs in the *Clostridiaceae* family as these are described as anaerobes and the incubations were performed under oxic conditions. Presumably in these incubations there were pockets of anoxia for the clostridia to thrive, such as in soil aggregates (Højberg *et al*., 1994). Soil clostridia have previously been described as N_2_‐fixers and as having a saccharolytic metabolism (Wiegel *et al*., 2006), which is consistent with them being detected as active diazotrophs in these incubations supplemented with carbon. Despite previous work on pure cultures suggesting that OTUs comprising as little as 0.001% of the community could be detected as differentially abundant in a SIP experiment (Aoyagi *et al*., 2015), we believe our threshold to be probably closer to 0.5%. As such, we propose that the identification of enriched OTUs for any nucleic acid‐based SIP should encompass not only log_2_ fold change (DESeq2) but also read count/OTU abundance and visual inspection of the data.

### Feasibility of using SIP‐Raman microspectroscopy for detecting ^15^N‐labelled cells

While the ^15^N Raman microspectroscopy method has been shown to work successfully in *E. coli* and potentially in other bacterial strains (Wang *et al*., 2013; Muhamadali *et al*., 2015), a comprehensive evaluation of the method encompassing (1) information on the occurrence of indicative peaks in species other than *E. coli;* (2) the ability to detect ^15^N incorporation at low levels across phylogenetically diverse species; and (3) the ability to predict ^15^N incorporation in unknown strains has not yet been performed. To address these open questions, we explored cellular Raman spectra for shifts with increasing ^15^N incorporation in a collection of phylogenetically diverse bacteria spanning five phyla: *Bacillus subtilis (Firmicutes)*, *Staphylococcus aureus (Firmicutes)*, *Pseudomonas putida* (*Gammaproteobacteria*), *Acidobacteriaceae* bacterium TAA 166 (*Acidobacteria*)*, Flavobacterium johnsoniae* (*Bacteroidetes*), *Nostoc* sp. (*Cyanobacteria*) and *Calothrix* sp. (Cyanobacteria).

**Figure 7:**
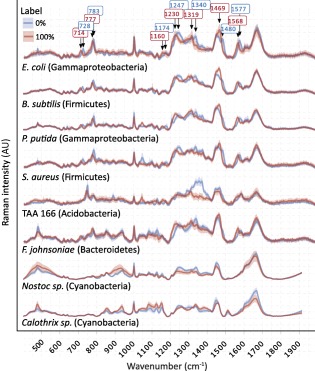
Differences in Raman spectra of unlabelled (blue lines) and ^15^N‐labelled (red lines) bacterial cells. Means (bold lines) and standard error (light bands) are depicted (*n* = ∼ 30). Numbers in boxes indicate peaks with significant shifts in response to ^15^N incorporation in *E. coli*. AU, arbitrary unit.

Comparison of the Raman spectra of unlabelled cells (grown in natural‐^15^N abundance medium) and fully labelled *E. coli* cells (grown in 99 at%‐^15^N media) showed shifts in peaks derived from N‐harbouring molecules (Fig. 7), as well as clustering of cell spectra according to labelling level in an NMDS analysis (data not shown), in accordance with previous reports (Muhamadali *et al*., 2015; Wang *et al*., 2016). Most notably, peaks at wavenumbers 728 (adenine breathing ring), 783 (O—P—O breathing, cytosine, uracil), 1174 (C—H in‐plane bending of tyrosine or phenylalanine), 1247 (amide III), 1340 (CH stretching of adenine), 1480 (purine bases) and 1577 cm ^−1^ (guanine, adenine ring stretching) shifted in position in the highly labelled cells, compared to unlabelled cells. However, only spectra originating from *B. subtilis*, *S. aureus* and *P. putida* displayed all of these aforementioned indicative peaks as observed in *E. coli* (Fig. 7). Strain TAA 166 lacked the peak at 728 cm^−1^ and *F. johnsoniae* lacked the peaks at 1174 and 1480 cm ^−1^. The two cyanobacterial strains, *Nostoc* sp. and *Calothrix* sp. lacked nearly all indicative peaks, with the exception of 1247, 1340 and 1480 cm^−1^ (Fig. 7).

Even though indicative peaks were not observed across all tested strains, we explored the possibility to detect ^15^N‐labelling of cells across this collection of phylogenetically diverse strains and enrichment levels (cells grown at 0, 10, 25, 50 and 100 at% ^15^N) using random forest classification models. A random forest constructed across all strains and enrichment levels showed an out‐of‐the‐box (OOB) error rate of about 20%; most measured cells fell in their correct category, however the model had difficulties distinguishing between 0% and 5% ^15^N labelling (Supporting Information Table S2). As most single‐cell investigations with Raman microspectroscopy seek to determine whether a cell is labelled or not, we constructed another random forest model with only two classes: unlabelled (0 and 5 at% ^15^N labelling) and labelled (10–100 at% ^15^N labelling). This model showed an OOB error rate of about 8.8% and a low false‐negative classification error rate (2%), but a high false‐positive classification error rate (21%; Table 2). However, the applicability of this model in experiments with complex microbial communities is limited since it only applies to the bacterial strains used to construct it. To test whether this approach can be used for predicting the labelling of an unknown bacterium, we constructed eight different random forest models, each time excluding one bacterial strain from the training set and then used the models to predict the labelling of the strain that was excluded from each model. The results of this model indicate that for some species (*E. coli*, *B. subtilis*, *P. putida* and *S. aureus*) the model performed well in predicting whether a cell is labelled or not, with sensitivity values ranging between 84.9% to 96.9% and specificity values ranging between 89.5% to 94.6%, while for other species (*F. johnsoniae*, *Acidobacteriaceae* bacterium TAA 166, *Nostoc* sp. and *Calothrix* sp.) the model performed rather poorly, with sensitivity values ranging between 32.0% to 91.4% and specificity values ranging between 22.2% to 83.6% (Table 3). A closer inspection of the origins of the classification errors showed that even for the bacterial species for which the models worked well (e.g., *E. coli*), the ability to predict labelling of cells that were grown in media containing ^15^N at levels of 10 and 25 at% was also poor. Taken together, the applicability of such a model might be limited to cells that are either unlabelled or highly labelled (approximately >50 at%; Supporting Information Table S3).

**Table 2:** Confusion matrix for the training data obtained from the Random forest model using all strains and two labelling levels.

Actual/predicted	Labelled	Unlabelled	Class. error
Labelled	885	20	0.02
Unlabelled	101	371	0.21

OOB estimate of error rate: 8.79%.

**Table 3:** Statistical measures of the performance of random forest models in predicting cell labelling.

	False pos. (%)	False neg. (%)	Sensitivity (%)^a^	Specificity (%)^b^
*E. coli*	7.1	4.3	94.2	94.6
*B. subtilis*	10.5	9.3	90.7	89.5
*P. putida*	10.0	13.6	84.9	91.1
*S. aureus*	7.8	2.9	96.9	92.7
*Acidobacteriaceae* bacterium TAA 166	39.1	35.7	91.4	20.9
*F. johnsoniae*	51.0	45.2	73.4	22.2
*Nostoc sp*.	35.3	43.2	32.0	83.6
*Calothrix* sp.	35.9	32.1	74.3	56.7

a Proportion of correctly identified positives.

b Proportion of correctly identified negatives.

As an alternative to standard Raman microspectroscopy, surface‐enhanced Raman scattering (SERS) in combination with SIP potentially offers enhanced sensitivity (Kubryk *et al*., 2015) and, therefore, the potential to better distinguish between ^15^N‐labelled and unlabelled cells. The quantitative detection of ^15^N incorporation in *E. coli* cells has already been demonstrated using SERS through a shift in an indicative peak at 728 cm^−1^, which is thought to be associated with adenine‐containing compounds (Kubryk *et al*., 2015; Wang *et al*., 2016). However, it has recently been shown that the source of the 728 cm^−1^ peak (as well as other prominent SERS spectra peaks) was due to secreted metabolites of the purine degradation pathway stemming from a stress response during sample preparation (Premasiri *et al*., 2016). This could limit the robustness of this method if cells are under different stress conditions and/or differential stress responses occur across species. Furthermore, the presence of these secreted metabolites is dependent on certain enzymatic pathways for purine catabolism, which are not universally distributed across microorganisms (Premasiri *et al*., 2016). Although these facets can greatly limit SERS applicability and reproducibility within and across different species, we still tested the applicability of ^15^N‐SERS on *E. coli* and *Acidobacteriaceae* bacterium TAA 166 cells. Freshly grown *E. coli* cells displayed an indicative peak at 728 cm^−1^, however, this peak was absent in cells of strain TAA 166 (Supporting Information Fig. S2). Moreover, the peak at 728 cm^−1^ in *E. coli* cells was only observed in fresh cells but not in formaldehyde‐fixed cells (which would not exhibit a stress response). *E. coli* cells that were stored for 5 days at 4°C exhibited a barely detectable 728 cm^−1^ peak (data not shown). In summary, it does not appear that SERS is a reliable alternative for detecting ^15^N‐labelled single cells.

## Conclusions

We have optimized and advanced our methodological toolbox and theoretical framework for investigating diazotrophs using stable isotope‐labelled ^15^N_2_. The ^15^N_2_ tracer assay for quantification of N_2_ fixation activity was more sensitive than ARA in the investigated soils with low diazotrophic activity. Additional sensitivity can be attained by targeting either RNA, nucleic acids (DNA/RNA) or proteins as compared to ‘bulk’ soil in ^15^N_2_‐tracer experiments and can also provide valuable information for downstream analysis of the diazotrophic microorganisms. Moreover, ^15^N‐RNA‐SIP is feasible for soil samples and using statistical modelling it is possible to reliably detect labelled diazotrophs. At this time, the use of Raman microspectroscopy and surface‐enhanced Raman spectroscopy to identify ^15^N‐enriched cells is not generally applicable across diverse microbial species. Alternatively, one can detect active diazotrophs in soil samples in a highly sensitive manner at the single‐cell level by NanoSIMS, as recently shown by Eichorst *et al*. (2015). Application of these methods has the potential of overcoming the difficulty in studying free‐living diazotrophs and of greatly advancing our knowledge of this ecologically important guild.

## Experimental procedure

### Comparison of ^15^N‐tracer assay with the acetylene reduction assay

#### Incubation conditions

To compare the performance of ARA and ^15^N_2_ tracer assays in detecting the activity of free‐living diazotrophs in soils, soil samples were obtained from a beech forest site near Klausen–Leopoldsdorf in Lower Austra, Austria (48°07′N 16°03′E) on 22 November 2013. The site has been previously described (Rasche *et al*., 2011). The soil is a dystric cambisol with a pH of 5.8, 6.6% soil organic carbon content, 0.6% total nitrogen content (both determined using dry combustion) and the water content was 55%. Three cores (8 cm diameter × 5 cm depth) were sampled and pooled after plant root and litter removal. Samples were stored at 4°C during transport to the laboratory. The soil was sieved through a 2‐mm sieve and homogenised. To stimulate N_2_‐fixation activity in the soil, two different carbon sources were added at the beginning of the experiment, at a concentration of 1.8 mg C g^−1^ soil (wet weight): artificial, N‐free root exudate mix (RE; modified from Griffiths *et al*., 1998 excluding amino acids) and fructose. In each vial (14 ml in volume) 1.7 g of sieved soil was incubated together with either 200 µl of carbon source solution or molecular‐grade water (Roth, Karlsruhe, Germany). Acetylene reduction and ^15^N_2_ tracer assays were performed on the same vials, in order to minimise the effect of soil heterogeneity on the results. Incubations were performed as follows: after weighing in soil and adding water and carbon sources, the vials were closed with a butyl‐rubber stopper (cleaned by boiling in water) and an aluminium crimp cap. The gas phase was either exchanged with a mixture of ^15^N_2_:He:O_2_ (40:40:20) (‘^15^N_2_’ samples) or the lab air was retained (‘control’ samples). Samples were incubated for 2 and 5 days in the dark. At each time point (2 and 5 days) an ARA was performed prior to sacrificing the vials for analysis of the soil using isotope ratio mass spectrometry (IRMS). Because of the limited headspace volume of the vials, gas samples could only be withdrawn once. Hence, vials of the first time point for the ARA experiment (T0 h) were prepared in parallel (see below). As a control for potential ethylene consumption by soil (Zechmeister‐Boltenstern and Smith, 1998), parallel vials were setup containing 0.05% acetylene (>99.6%, Air Liquid, Paris, France) and 25 ppm of ethylene (100 ppm, Sigma‐Aldrich, St. Louis, MO, USA) in the headspace and the soil was amended with ${\text{NH}}_{4}^{+}$‐N (1.2 mg g^−1^) and C‐sources (fructose or RE), and incubated under the same conditions as described above. Then ARA measurements were performed as described below. A liquid culture of *Anabaena torulosa* was used as a positive control for the ARA experiments. The culture was grown in a liquid N‐free Jüttner medium (Jüttner, 1976) for 1 week at about 20°C under 12 h diurnal cycle using artificial light, prior to measuring N_2_ fixation using ARA.

#### Acetylene reduction assay

ARA was performed as previously described (Unkovich *et al*., 2008), using 10% acetylene (C_2_H_2_). Measurements of ethylene (C_2_H_4_) production were performed at t_0_ and after four hours. All gas samples were stored in evacuated 3 ml exetainer vials until measurement. For the positive control, 2 ml of *Anabaena torulosa* liquid culture was incubated in 14 ml glass vials for 4 h under artificial light at 20°C. The ARA set up was identical to the soil samples, with time 0 and 4 h samples being taken from parallel vials. Gas samples were then analysed via GC (TRACE GC Ultra equipped with a Porapak R column and an FID detector, Thermo Fisher Scientific, Waltham, MA, USA). Based on a standard curve, we assumed reliable linear quantification at about 5 ppmv ethylene.

#### ^15^N tracer assay

The ^15^N/^14^N isotope ratios were determined by elemental analysis‐isotope ratio mass spectrometry (EA‐IRMS on a EA 1110; CE Instruments, Milan, Italy, coupled to a Finnigan MAT DELTA Plus IRMS; Thermo Fisher Scientific). Measurements were performed against atmospheric air as a standard, and the results are expressed in the delta notation:

$$\delta^{15}N\left(‰\right)=\left[\frac{{\left({}^{15}N/{}^{14}N\right)}_{sample}}{{\left({}^{15}N/{}^{14}N\right)}_{atmosphere}}-1\right]\times 1000$$

#### Comparison between ARA and ^15^N tracer assay

The amount of ethylene produced based on the measured ^15^N enrichment of bulk soil was estimated as follows: soil N content (µmol g^−1^ dw) was multiplied by atom‐percent‐excess (APE) of ^15^N of incubated soil, and divided by the incubation time (hours). APE was calculated by subtracting ^15^N natural abundance controls (atom %) from ^15^N labelled samples (atom %). The resulting APE indicates the amount of ^15^N incorporated into the sample by diazotrophs. Based on this ^15^N incorporation rate and assuming a theoretical molar conversion factor of 3:1 acetylene to N (Hardy *et al*., 1973), a reduction rate in µmol C_2_H_2_ g^−1^ h^−1^ was calculated and converted to ppm C_2_H_2_ reduced per incubation vial. The incorporation rate of ^15^N per gram of soil was calculated and expressed as a rate (µmol ^15^N_2_ g^1^ h^−1^).

### Bulk soil analysis

Bulk soil was dried at 60°C for 48 h. The samples were homogenised in a ball mill (MM2000, Retsch, Haan, Germany) by shaking at an amplitude of 90% for 10 min with two metal beads (ø 2 mm). Of the milled soil, 5–8 mg was transferred into tin capsules (IVA Analysentechnik, Düsseldorf, Germany) and analysed by EA‐IRMS as described above.

### Tracing ^15^N incorporation into biomolecules

#### Incubation conditions

For tracing ^15^N incorporation into different biomolecules, nine vials containing soil and ^15^N_2_ gas in the headspace and four control vials were incubated similarly to the way described above: glass vials (approx. 14 ml) contained 3 g of sieved soil and 320 µl of artificial root exudate mixture (see above). The gas phase was either exchanged with a mixture of ^15^N_2_:He:O_2_ (40:40:20) or the lab air was retained in the control samples. Vials containing ^15^N_2_ gas were incubated for 1, 3 and 14 days in triplicates while control samples were incubated for 0 and 14 days, before being sacrificed. The soil was aliquoted and stored at −80°C until analysis. In addition, aliquots from unincubated sieved soil were stored at −80°C to serve as t_0_ controls.

#### Protein extractions

Proteins were extracted from soil following Schneider *et al*. (2012), except for the grinding step, which was performed by transferring 1 g of soil to a lysing matrix E tube (MP BioMedicals California, USA) and snap‐freezing it in liquid N_2_. Then the sample was processed by beat beating (1 min, 6.5 m/s, with dry ice in a FastPrep‐24 Instrument; MP‐Biomedicals, Santa Ana, CA, USA), four consecutive times. Quantification was performed using the Ninhydrin assay (following 24 h hydrolisation at 100°C with 6 N HCl) (Starcher, 2001), with BSA (20 mg ml^−1^; Thermo Fisher Scientific) as a standard. For IRMS analysis, aliquots of 0.5–0.9 mg protein extract were transferred to flat bottom smooth tin capsules (IVA Analysentechnik), dried at 60°C and analysed by EA‐IRMS as described above.

#### DNA and RNA extraction and purification

Nucleic acids (NA) were extracted from 0.4 g of soil using bead‐beating in the presence of a CTAB buffer and phenol, according to a previously published extraction protocol (Angel, 2012). Following extraction, samples were purified using OneStep™ PCR Inhibitor Removal Kit (Zymo, Irvine, CA, USA) and DNA was quantified using Quant‐iT™ PicoGreen® (Thermo Fisher Scientific). From this extract, 100 µl were used for RNA removal: 10 µl of RNase I (Thermo Fisher Scientific) was added and the sample was incubated at 37°C for 1 hour. Then, the DNA was purified from the digested RNA using QIAquick PCR Purification Kit (Qiagen, Venlo, Netherlands). The DNA was eluted in 50 µl of nuclease free water, quantified using the Quant‐iT™ PicoGreen® assay and stored at −20°C until analysis. RNA was purified by digesting 10–42 µl of NA solution (1–3 µg of DNA) using Turbo DNase (Thermo Fisher Scientific), followed by purification using GeneJET RNA Cleanup and Concentration Micro Kit and elution in RNase Storage Solution (both from Thermo Fisher Scientific). The RNA yield was quantified using the Quant‐iT™ RiboGreen® assay (Thermo Fisher Scientific).

For IRMS analysis, 2–5 µg of DNA, RNA or NA was transferred to flat bottom smooth tin capsules (IVA Analysentechnik), spiked with proline‐sucrose mixture (containing 1.11 µg N with a δ^15^N 0.11‰) and analysed by EA‐IRMS. Calculations of δ^15^N for DNA, RNA and NA were done as follows, based on a two‐source mixing model:

$$\delta^{15}N_{\text{RNA}}=\;\left(\delta^{15}N_{\text{total}}\times {\;N}_{\text{total}}–\;\delta^{15}N_{\text{spike}}\times {\;N}_{\text{spike}}\right)/\left(N_{\text{total}}–{\;N}_{\text{spike}}\right),$$

where δ^15^N and N refer to the isotopic composition and N amount of measured fractions, and spike to the added proline‐sucrose mixture and total to the combined signal of spike plus sample.

### ^15^N‐RNA‐SIP

#### Soil samples and incubation conditions

For validating the ability to separate ^15^N‐labelled and unlabelled RNA, cultures of *Flavobacterium johnsoniae* and *Pseudomonas putida* were grown in media containing ^14^NH_4_, (both strains) and ^15^NH_4_ (*F. johnsoniae only)*. Cultures were grown as described below under ‘Testing the applicability of ^15^N‐based SIP‐Raman’, except that the cultures were not fixed in paraformaldehyde (PFA), but directly used for RNA extraction and density gradient centrifugation. For testing the applicability of ^15^N‐RNA‐SIP for soil samples, two grams of homogenised soil samples from Klausen–Leopoldsdorf were incubated on the day of sampling as described above under ‘Tracing ^15^N incorporation into biomolecules’. For the SIP experiment¸ vials containing ^15^N_2_ gas were incubated for 3, 7 and 21 days and the control vials for 21 days. All incubations were done in the dark, at room temperature. For the 21 d incubations, 2 ml of O_2_ was injected to the vials weekly. Following incubation, the vials were sacrificed and the soil was frozen at −80°C until further analysis.

#### DNA and RNA stable isotope probing

DNA (ca. 2.4 µg) was subjected to isopycnic gradient centrifugation in a solution of caesium chloride (CsCl, Sigma Aldrich), HiDi formamide (Thermo Fisher Scientific) and buffer (0.1 M Tris‐HCl at pH 8.0, 0.1 M KCl and 1 mM EDTA, all from Sigma Aldrich) as described previously (Neufeld *et al*., 2007), but with the final density adjusted to 1.69 g ml^−1^ (Buckley *et al*., 2007b). RNA (ca. 350 ng) was subjected to isopycnic gradient centrifugation in a solution of caesium trifluroacetate (CsTFA, GE Healthcare, Little Chalfont, UK), HiDi formamide (Thermo Fisher Scientific) and buffer (0.1 M Tris‐HCl at pH 8.0, 0.1 M KCl and 1 mM EDTA) as described previously (Dumont *et al*., 2011). Following centrifugation, the DNA‐ or RNA‐gradient solutions were fractioned into 20 fractions of equal volume (Lueders *et al*., 2003). From each DNA‐SIP gradient, three fractions corresponding to densities between 1.725 and 1.742 g ml^−1^ were used for downstream analysis. DNA was recovered from fractions using precipitation in a Polyethylene glycol (PEG) solution (30% PEG, 1.6 M NaCl) in the presence of 20 µg glycogen (RNA grade, Thermo Fisher Scientific) and subjected to a secondary isopycnic gradient centrifugation in a CsCl solution containing 10 mg ml^−1^ of bis‐benzimide (Sigma‐Aldrich) as a DNA intercalating agent (Buckley *et al*., 2007a). Following the secondary centrifugation, DNA was recovered from fractions corresponding to densities between 1.650 ± 0.005 and 1.750 ± 0.005 g ml^−1^ as described above and used directly as a template for downstream molecular analysis. From each RNA‐SIP gradient, fractions corresponding to densities between 1.745 ± 0.005 and 1.835 ± 0.005 g ml^−1^ were used for downstream analysis. From each fraction, the RNA was ethanol‐precipitated in the presence of 20 µg of GlycoBlue Coprecipitant and 0.1 volume 5 M ammonium acetate and resuspended in 10 µl of RNA storage solution (all solutions from Thermo Fisher Scientific). Complete, random cDNA was synthesized from 10 µl of fractionated RNA using SuperScript^TM^ III reverse transcriptase and 0.5 µg µl^−1^ of random hexamer primers (both from Thermo Fisher Scientific), as described by the manufacturer. Validation of DNA or RNA enrichment with heavy isotopes was done by quantifying the number of bacterial rRNA copies in each fraction, using a qPCR assay described below.

#### qPCR quantification of SIP fractions

Quantification of rRNA copies in each SIP fraction was done using qPCR on an iCycler thermocycler equipped with a MyiQ detection system (Bio‐Rad, Hercules, CA, USA) and the data were processed using iQ5 Optical System software (Bio‐Rad). Results were expressed as a fraction of the total number of copies. Primers BAC338F – BAC805R together with the dual‐labelled probe BAC516F (Yu *et al*., 2005) were employed for quantification. Each reaction was 20 µl in volume and contained the following mixture: 10 µl of iQ™ Supermix (Bio‐Rad), 4 mM MgCl_2_, 0.5 mM of each primer, 0.2 mM of the dual‐labelled probe (Thermo Fisher Scientific) and 2 µl of template. For both assays, the programme used was: 94°C for 5 min, followed by 40 cycles of 94°C for 30 s and 62°C for 60 s for annealing, extension and signal acquisition. The standards used to determine the copy numbers in the samples were cloned fragments of the 16S rRNA gene of *E. coli*.

#### PCR amplification and sequencing

Sequencing of the DNA or cDNA from the SIP fractions was done using multiplexed barcoded amplicon sequencing on an Illumina MiSeq platform (Illumina, San Diego, CA, USA), as described previously (Herbold *et al*., 2015). Primers 515F_mod – 806R_mod targeting about 292 bp in the V3 and V4 regions of the 16S rRNA gene in bacteria and archaea (Apprill *et al*., 2015; Parada *et al*., 2015) were used. A list of the barcodes used for sequencing can be found in Supporting Information Table S1. First‐step PCR amplifications were done in triplicates of 25 µl, each using the following mixture: 5 µl of 10 × DreamTaq Green Buffer, 2 mM MgCl_2_, 0.2 mM of each nucleotide dNTP mixture, 0.08 µg µl^−1^ of BSA, 0.625 U of DreamTaq Green DNA Polymerase (all from Thermo Fisher Scientific) and 0.25 µM of each headed primer (Thermo Fisher Scientific) and 1 µl of DNA or cDNA template. The following program was used for amplification: 94°C for 5 min followed by 22 cycles (for high‐template fractions between 1.745 ± 0.005 and 1.795 ± 0.005 g ml^−1^ in the RNA gradients and between 1.691± 0.005 and 1.740 ± 0.005 g ml^−1^ in the DNA gradients) or 24 cycles (for all other fractions) of 94°C for 30 s, 52°C for 45 s and 72°C for 45 s, and a single step of final elongation at 72°C for 10 min. Following PCR amplification, samples were purified using ZR‐96 DNA Clean‐up Kit™ (Zymo) and 3 µl from the purified sample was used for a second PCR reaction, which was 50 µl in volume and contained the following mixture: 5 µl of 10 × DreamTaq Green Buffer, 2 mM MgCl_2_, 0.2 mM of each nucleotide dNTP mixture, 0.08 µg µl^−1^ of BSA, 1.875 U of DreamTaq Green DNA Polymerase (all from Thermo Fisher Scientific) and 0.4 µM of a headed‐barcode primer. The following program was used for amplification: 94°C for 5 min followed by 8 cycles of 94°C for 30 s, 52°C for 45 s and 72°C for 45 s, and a single step of final elongation at 72°C for 10 min. Following this barcoding‐PCR step, the samples were purified using ZR‐96 DNA Clean‐up Kit™ (Zymo), quantified using Quant‐iT™ PicoGreen® dsDNA Assay Kit (Thermo Fisher Scientific) on a Tecan Safire plate reader (Tecan) and pooled in equimolar amounts of 20 × 10^9^ copies per sample. Library preparation and sequencing services were provided by Microsynth (Balgach, Switzerland). The library was prepared by adaptor ligation and PCR using the TruSeq Nano DNA Library Prep Kit (Illumina) according to the TruSeq nano protocol (Illumina, FC‐121–4003), but excluding the fragmentation step. Sequencing was performed on a MiSeq platform (Illumina). The MiSeq was run in the 2 × 300 cycle configuration using the MiSeq Reagent kit v3 (Illumina). The raw sequence data were deposited into the NCBI Short Read Archive under BioProject accession number PRJNA369325.

#### Sequence data processing

Paired reads generated by the MiSeq platform were clustered into OTUs as previously described (Herbold *et al*., 2015). Briefly, paired reads were assembled into contigs using QIIME's join_paired_ends.py (Caporaso *et al*., 2010). Then, sequences were chimera‐checked and clustered using the UPARSE pipeline (Edgar, 2013). First contigs were dereplicated with the ‐derep_fullength command and singleton unique sequences were removed. OTU centroids were then determined with the ‐cluster_otus command (using 3% radius). Abundances of OTUs were determined by mapping the filtered contigs (prior to dereplication) to OTU centroids using the ‐usearch_global command (using 97% identity). OTU representatives were classified using mothur's implementation of a Naïve Bayesian sequence classifier (Wang *et al*., 2007; Schloss *et al*., 2009) against the SILVA 115 SSU NR99 database at a confidence cutoff of 50% (Quast *et al*., 2013). Beta‐diversity analysis of the communities in the SIP fractions was done in R (V3.3.1; R Core Team, 2016). Principal coordinates analysis based on Morisita–Horn dissimilarities of OTU counts (normalised using DESeq2::estimateSizeFactors; (Horn, 1966; Anders and Huber, 2010) was computed using vegan::capscale and plotted in ggplot2.

#### Identification of enriched OTUs from labelled RNA or DNA

To identify which bacteria or archaea incorporated ^15^N into their nucleic acids and therefore performed N_2_ fixation, we employed a similar approach to the one described by Pepe‐Ranney *et al*. (2016), with modifications. Based on the fact that unlike in DNA‐SIP in RNA‐SIP the G + C content of the molecules does not influence their positioning along the density gradient (see Dumont *et al*., 2011 and this study), we assumed that in a density gradient with unlabelled RNA only: (1) most of the RNA will concentrate in fractions corresponding to densities between about 1.760 and 1.786 g ml^−1^ (i.e., the ‘light’ fraction) while some RNA will be present throughout the gradient and (2) that the proportions of each RNA sequence (represented as an OTU) will not vary throughout the gradient. In contrast, if some of the RNA is labelled it will show a higher than average proportion in the ‘heavy’ fractions of the gradient (corresponding to densities of ca. 1.785 to 1.820 g ml^−1^) compared to the ‘light’ fractions (corresponding to densities of ca. 1.740 to 1.785 g ml^−1^). This way of detecting differentially abundant OTUs is advantageous to comparing ‘heavy’ fractions from labelled and control gradients, since this way one avoids a potential ‘bottle effect’ (i.e., the stochastic differences between vials incubated under the same experimental conditions), which might lead to detecting false positives. The same approach can also be applied to DNA‐SIP if the problem of G + C content bias has been overcome (such as with the method employed here), except that in this case enrichment with labelled DNA should be expected in the ‘light’ fraction. The main challenge in identifying these enriched OTUs is to find a statistically robust framework, which can account for read‐depth differences in the sequence libraries [i.e., the different SIP fractions in this case, see McMurdie and Holmes (2014) for an extended discussion on the matter]. To this end we used the DESeq2 pipeline (Love *et al*., 2014), which was originally designed to test for differential expression in RNA‐Seq experiments. We used this pipeline for analysing the ‘OTU differential abundance’ (McMurdie and Holmes, 2014) between the ‘labelled’ fractions (i.e., ‘heavy’ fractions in our RNA‐SIP, but ‘light’ fractions in the DNA‐SIP). The pipeline was modified to analyse our SIP data as follows: (1) sparse OTU filtering: to speed computation time, OTUs with a total count of less than 10 reads across all samples and OTUs which appear in less than 20% of the samples were removed prior to analysis. (2) the DESeq2 pipeline was then applied to each gradient separately (including the control gradients), more specifically: (a) sample‐specific ‘size‐factors’ were estimated to fit ‘gene‐specific’ (OTU) means, (b) ‘gene‐wise’ (OTU) dispersion was estimated and (c) count data were modelled by fitting a negative binomial distribution using the fitted mean and dispersion. (3) For each gradient, a comparison of OTU differential abundance between ‘labelled’ and ‘unlabelled’ fractions of the gradient was performed using a one‐sided Wald test, employing a false discovery rate of 0.1 and a 0.25 log_2_ fold change (ca. 1.19 fold enrichment). Correction of *P*‐values for multiple testing was done using the Benjamini–Hochberg method (Benjamini and Hochberg, 1995). (4) the results of the modelling were confirmed by: (a) comparing the results of modelling of the labelled gradients with those of the unlabelled gradients, and (b) plotting the normalised read‐number distribution of each OTU along the gradient fractions to visually spot potential false positives. In addition, for the sake of comparison we also performed the analysis in a similar fashion to Pepe‐Ranney *et al*. (2016), namely, by comparing only the fractions where labelled templates are expected to concentrate (i.e., ‘heavy’ fractions in the RNA‐SIP and ‘light’ fractions in the DNA‐SIP) between the labelled and control gradients after 21 d incubation.

### Testing the applicability of ^15^N‐based SIP‐Raman microspectroscopy

#### Cultures used and cultivation conditions

To test the applicability of Raman microspectroscopy in detecting ^15^N‐labelled cells, eight bacterial strains were grown with various levels of ^15^N enrichment of NH_4_ in the growth media. The following five bacterial species were grown in a VSB medium (pH 7; Eichorst *et al*., 2007), aerobically, at 37°C: *Escherichia coli*, *Bacillus subtilis*, *Pseudomonas putida, Staphylococcus aureus* and *Flavobacterium johnsoniae*, while *Acidobacteriaceae* bacterium TAA 166) (Eichorst *et al*., 2007) was grown in a VSB medium (pH 6). In both cases cultures were grown with 20 mM ${\text{NH}}_{4}^{+}$ as a sole nitrogen source. In addition, two cyanobacterial species (*Nostoc* sp. and *Calothrix* sp.) were grown in BG11 medium (Stanier *et al*., 1971), aerobically, at 37°C with 20 mM ${\text{NO}}_{3}^{-}$ as a sole nitrogen source. Labelling of approximately 0 (natural abundance), 5, 10, 25, 50 and 100 at% ^15^N was achieved by mixing stock solutions of ^15^N ‐ ${\text{NH}}_{4}^{+}$ or ^15^N‐ ${\text{NO}}_{3}^{-}$ (NH_4_Cl >99 or NaNO_3_ >98 at% ^15^N, Cambridge Isotope Laboratories) with natural ^15^N abundance stocks of ${\text{NH}}_{4}^{+}$ or ${\text{NO}}_{3}^{-}$ at varying proportions. An inoculum of each culture was transferred three consecutive times during logarithmic phase to ensure uniform labelling of the cells. Then, a millilitre of culture was centrifuged for 1 min at 10 000 r.p.m., cells were washed with phosphate‐buffered saline (PBS; 130 mM NaCl, 5% phosphate buffer; pH 7.0) and suspended in a solution of 1% PFA in PBS for fixation (1 h, room temperature). Following fixation, the cells were again centrifuged, the pellet was washed once in PBS, centrifuged again and finally resuspended in 1:1 solution of EtOH:PBS and stored at −20°C until measurement.

For surface‐enhanced Raman scattering (SERS) analysis, cultures of *E. coli* and *Acidobacteriaceae* bacterium TAA 166 were grown overnight as described above using unlabelled media. Following centrifugation and washing of the cells once with Milli‐Q (MQ) water (Millipore, Billerica, MA, USA) the cells were pelleted again. Coating of the cells with silver colloids was done as described previously (Kubryk *et al*., 2016).

#### Raman microspectrosopy

To obtain the Raman spectra of the aforementioned cultures, 2 µl of fixed cells was spotted on aluminum‐coated slides (Al136; EMF Corporation, Ithaca, NY, USA), dried for several minutes at 48°C, briefly washed in MQ water to remove excess buffer salts and then dried again. Raman spectra of single microbial cells in these cultures were acquired using a LabRAM HR800 confocal Raman microscope (Horiba Jobin‐Yvon, Kyoto, Japan) equipped with a 532‐nm neodymium‐yttrium aluminium garnet laser and either 300 or 600 grooves mm^−1^ diffraction grating as previously described (Haider *et al*., 2010). Spectra were acquired in the range of 400–1915 cm^−1^.

Because of the presence of photosynthetic pigments in the cyanobacterial cultures, attempting to measure Raman spectra using the above‐stated conditions resulted in masking of the cell‐spectra by intense peaks resulting from the pigments. Therefore, cells of the cyanobacterial cultures were not measured in a dry state but instead in water on a calcium fluoride slide. Raman spectra of single microbial cell for these cultures were acquired using the same conditions as described above, except that prior to data acquisition the cells were bleached by the laser for several seconds until a stable background signal was reached.

#### Analysis of Raman spectra and statistical modelling

All preprocessing steps and downstream statistical analyses were done using R (V 3.3.1; R Core Team, 2016). Raman spectra (ranging between 400 and 1914 cm^−1^) were confirmed to be correctly aligned by tracing the peak indicative of phenylalanine at 1003 cm^−1^. Spectra were then preprocessed as described previously (Bocklitz *et al*., 2011). First, a background correction was done using polynomial fitting (sixth degree polynomial, with a tolerance of 0.01 and maximum 100 iterations; R package ‘baseline’ (Lieber and Mahadevan‐Jansen, 2003; Liland and Mevik, 2015). Thereafter, each spectrum was normalized to the sum of its absolute spectral intensity. Classification of ^15^N‐labelled cells was done by constructing random forest models [R package ‘randomForest’; (Breiman, 2001; Liaw and Wiener, 2002)]. For each model, the data representing labelling levels of 0, 5, 10, 25, 50 and 100 at% of all bacterial strains but the one tested were used as a training set while the entire dataset of the tested strain was used as a test set. The results of this classification are presented in a confusion matrix, where true class labels are compared with the predicted ones. Using these confusion matrices, we calculated the ratio of false positives, false negatives, the sensitivity and specificity of each model. The rate of false positives was calculated as the ratio of the number of cells falsely predicted to be labelled divided by the total number of cells predicted to be labelled (false and correct). The rate of false negatives was calculated as the ratio of the number of cells falsely predicted to be unlabelled divided by the total number of cells predicted to be unlabelled (false and correct). The sensitivity of the model (the chance of correctly identifying a labelled cell) was calculated as the ratio of the number of correctly predicted labelled cells divided by the total number of labelled cells (correctly or incorrectly predicted), while the specificity of the model (the chance of correctly identifying an unlabelled cell) was calculated as the ratio of the number of correctly predicted unlabelled cells divided by the total number of unlabelled cells (correctly or incorrectly predicted).

## Supporting information

Additional Supporting Information may be found in the online version of this article at the publisher's web‐site:

**Supporting information.** Separating ^15^N‐labelled RNA and DNA from pure cultures.
**Supplementary Table 1**. Barcodes used for sequencing.
**Supplementary Table 2**. Confusion matrix for the training data obtained from a Random forest model using all strains and labelling levels.
**Supplementary Table 3**. Confusion matrix for classifying *E. coli* test data obtained from a Random forest model using all strains but *E. coli* and all labelling levels.
**Supplementary Fig. 1.** Proportion of bacterial 16S rRNA copies recovered from each of the SIP gradient fractions from pure cultures. Values on the Y‐axis represent the proportion of the RNA copies out of the total number of RNA copies of the entire gradient. A. RNA‐SIP (CsTFA density gradients) of each culture type run independently. B. RNA‐SIP of a mixture of the two cultures. C. Primary DNA‐SIP (CsCl density gradients) of the two cultures.
**Supplementary Fig. 2**. SERS spectra of unlabelled *E. coli* and *Acidobacteriaceae* bacterium TAA 166 cells. Means (bold lines) and standard error (light bands) are depicted (*n* = ∼10). Number label indicates the position of the peak associated with adenine compounds.
