# Supplementary material for: Application of stable‐isotope labelling techniques for the detection of active diazotrophs
Source: Environ Microbiol. 2017 Dec 15;20(1):44–61. doi: 10.1111/1462-2920.13954 (PMC5814836; doi:10.1111/1462-2920.13954)
Supplement: Supplementary file 1 — Supporting information. Separating 15N‐labelled RNA and DNA from pure cultures. Supplementary Table 1. Barcodes used for sequencing. Supplementary Table 2. Confusion matrix for the training data obtained from a Random forest model using all strains and labelling levels. Supplementary Table 3. Confusion matrix for classifying E. coli test data obtained from a Random forest model using all strains but E. coli and all labelling levels. Supplementary Fig. 1. Proportion of bacterial 16S rRNA copies recovered from each of the SIP gradient fractions from pure cultures. Values on the Y‐axis represent the proportion of the RNA copies out of the total number of RNA copies of the entire gradient. A. RNA‐SIP (CsTFA density gradients) of each culture type run independently. B. RNA‐SIP of a mixture of the two cultures. C. Primary DNA‐SIP (CsCl density gradients) of the two cultures. Supplementary Fig. 2. SERS spectra of unlabelled E. coli and Acidobacteriaceae bacterium TAA 166 cells. Means (bold lines) and standard error (light bands) are depicted (n = ∼10). Number label indicates the position of the peak associated with adenine compounds. [file EMI-20-44-s001.pdf]

Supporting information

Separating ^{15}N-labelled RNA and DNA from pure cultures

To test the feasibility of ^{15}N-RNA-SIP, particularly when the RNA originates from organisms with stark differences in their G+C content, unlabelled and 100% ^{15}N-labelled RNA from the low G+C bacterium *F. johnsoniae* (genomic G+C content = 34%) and unlabelled RNA from the high G+C bacterium *P. putida* (genomic G+C content = 62%) were subjected to ultracentrifugation in CsTFA gradients. Following centrifugation, fraction collection, precipitation and cDNA synthesis, the SSU rRNA copy numbers in each fraction were quantified. When loaded separately on a CsTFA density gradient, ^{15}N-labelled RNA from *F. johnsoniae* concentrated mostly in the 'heavy' fractions of the gradient (between 1.78 and 1.82 g ml^{-1}) while unlabelled RNA concentrated mostly in the 'light' fractions of the gradient (between 1.76 and 1.78 g ml^{-1}). Unlabelled RNA from *P. putida* also concentrated in the 'light' fractions of the gradient despite having a much higher G+C content compared to *F. johnsoniae* (Supplementary Fig. 1A). Also when mixed together, a ^{15}N-labelled RNA from G+C-poor bacteria could clearly be separated from the unlabelled RNA of a G+C-rich bacteria (Supplementary Fig. 1B). In contrast, as previously demonstrated by Buckley and colleagues (2007), labelled DNA of *F. johnsoniae* did not separate from unlabelled DNA of *P. putida*; both concentrated in the fractions between 1.72 and 1.74 g ml^{-1} (Supplementary Fig. 1C). In addition, despite previous concerns of strong interactions between RNA molecules (Addison *et al.*, 2010), which could have emerged from overloading the density gradients with RNA or from the specific run conditions, we did not observe much interaction between labelled and unlabelled RNA molecules, as evident by the similar positioning of the labelled and unlabelled peaks when the differently labelled RNA samples were run separately or together.

References

Addison, S.L., McDonald, I.R., and Lloyd-Jones, G. (2010) Stable isotope probing: Technical considerations when resolving ^{15}N-labeled RNA in gradients. *J. Microbiol. Methods* **80**: 70–75.

Buckley, D.H., Huangyutitham, V., Hsu, S.-F., and Nelson, T.A. (2007) Stable isotope probing with ^{15}N achieved by disentangling the effects of genome G+C content and isotope enrichment on DNA density. *Appl. Environ. Microbiol.* **73**: 3189–3195.

Supplementary Table 1. Barcodes used for sequencing

Sample	Template	Fraction	Barcode	Template	Fraction	Barcode
KLD3 Fruc 3 15N I	RNA	4	ACGTGTTG	DNA	1	CGATCCTT
KLD3 Fruc 3 15N I	RNA	5	ATCCAACG	DNA	2	GTCTCACT
KLD3 Fruc 3 15N I	RNA	6	TCTGGTCT	DNA	3	CATCACCT
KLD3 Fruc 3 15N I	RNA	7	ACGTGAAG	DNA	4	ACTGACTG
KLD3 Fruc 3 15N I	RNA	8	TCACGAGT	DNA	5	CAGATCTG
KLD3 Fruc 3 15N I	RNA	9	AGTGGAGT	DNA	6	GACACTGT
KLD3 Fruc 3 15N I	RNA	10	TTGGATCG	DNA	7	TGGTTCCT
KLD3 Fruc 3 15N I	RNA	11	CACAGTGT	DNA	8	CCAACCTT
KLD3 Fruc 3 15N I	RNA	12	AGTCAGAG	DNA	9	ACGACAAG
KLD3 Fruc 3 15N I	RNA	13	ATCGATCG	DNA	10	CTACACCT
KLD3 Fruc 3 15N I	RNA	14	CCATATGG	DNA	11	GCTACCTT
KLD3 Fruc 3 15N I	RNA	15	GTCTACAG	DNA	12	CAGTAGAG
KLD3 Fruc 3 15N I	RNA	16	CCTACCAT	DNA	13	TACGTACG
KLD3 Fruc 3 15N I	RNA	17	GCTTCGTT	DNA	14	CCTATAGG
KLD3 Fruc 3 15N I	RNA	18	CGAACGTT	DNA	15	TGGACAAG
KLD3 Fruc 7 15N I	RNA	4	GACTAGAG	DNA	1	TCACCACT
KLD3 Fruc 7 15N I	RNA	5	GAGAGACT	DNA	2	CGATGGAA
KLD3 Fruc 7 15N I	RNA	6	AAGGCGAT	DNA	3	GTGTTCAG
KLD3 Fruc 7 15N I	RNA	7	TGGTTGGT	DNA	4	CTAGTCCT
KLD3 Fruc 7 15N I	RNA	8	TCTGACAG	DNA	5	GCCGTAAT
KLD3 Fruc 7 15N I	RNA	9	CCTATTCG	DNA	6	AGACACAG
KLD3 Fruc 7 15N I	RNA	10	CTTCGAAG	DNA	7	CAAGACCT
KLD3 Fruc 7 15N I	RNA	11	AGTCACTG	DNA	8	ACAGGAGT
KLD3 Fruc 7 15N I	RNA	12	GAGTCTGT	DNA	9	GGAACCAA
KLD3 Fruc 7 15N I	RNA	13	CACTACAG	DNA	10	TGTGTGTG
KLD3 Fruc 7 15N I	RNA	14	CGTTCGTT	DNA	11	AACCAAGG
KLD3 Fruc 7 15N I	RNA	15	GTACCTAG	DNA	12	TCTCGACT
KLD3 Fruc 7 15N I	RNA	16	CAAGAGGT	DNA	13	CGAATAGG
KLD3 Fruc 7 15N I	RNA	17	GACAGTCT	DNA	14	TCTGGAGT
KLD3 Fruc 7 15N I	RNA	18	TAATGCCG	DNA	15	TGAGACAG

Sample	Template	Fraction	Barcode	Template	Fraction	Barcode
KLD3_Fruc_21d_15N_I	RNA	4	TCGTCAAG	DNA	1	TCTGAGTG
KLD3_Fruc_21d_15N_I	RNA	5	TGTCGAGT	DNA	2	TACCGGAT
KLD3_Fruc_21d_15N_I	RNA	6	CATCGTAG	DNA	3	ACAGTGAG
KLD3_Fruc_21d_15N_I	RNA	7	GAAGTCCT	DNA	4	TACGATCG
KLD3_Fruc_21d_15N_I	RNA	8	TTGGTTGG	DNA	5	AGACGTCT
KLD3_Fruc_21d_15N_I	RNA	9	GTCTAGTG	DNA	6	TACCAACG
KLD3_Fruc_21d_15N_I	RNA	10	GTTGTGGT	DNA	7	CTCAAGTG
KLD3_Fruc_21d_15N_I	RNA	11	CGTACCTT	DNA	8	CTCACACT
KLD3_Fruc_21d_15N_I	RNA	12	ATCCGGAT	DNA	9	CAGACTGT
KLD3_Fruc_21d_15N_I	RNA	13	GTCTCTGT	DNA	10	GGATATGG
KLD3_Fruc_21d_15N_I	RNA	14	ATCCTAGG	DNA	11	GTGAGTCT
KLD3_Fruc_21d_15N_I	RNA	15	ACTCCACT	DNA	12	AGGAGAAG
KLD3_Fruc_21d_15N_I	RNA	16	GTCACTCT	DNA	13	GTGACACT
KLD3_Fruc_21d_15N_I	RNA	17	TCGATCCT	DNA	14	TGTGACTG
KLD3_Fruc_21d_15N_I	RNA	18	TGTCTGAG	DNA	15	CAACCTAG
KLD3_Fruc_21_14N_I	RNA	4	ACTCTGAG	DNA	1	GATCGTTG
KLD3_Fruc_21_14N_I	RNA	5	TGTCCTGT	DNA	2	TGTGTCAG
KLD3_Fruc_21_14N_I	RNA	6	TCGTCTTG	DNA	3	CTTCCATG
KLD3_Fruc_21_14N_I	RNA	7	AAGGATCG	DNA	4	ACTCTCTG
KLD3_Fruc_21_14N_I	RNA	8	CTGAGTGT	DNA	5	CTGTTCTG
KLD3_Fruc_21_14N_I	RNA	9	TAGGTAGG	DNA	6	TTCGATGG
KLD3_Fruc_21_14N_I	RNA	10	CCATCCAT	DNA	7	GATCTCCT
KLD3_Fruc_21_14N_I	RNA	11	GATGGATG	DNA	8	GACTGTGT
KLD3_Fruc_21_14N_I	RNA	12	AGGATGGT	DNA	9	GAGAGTGT
KLD3_Fruc_21_14N_I	RNA	13	CTCTACTG	DNA	10	CATCGATG
KLD3_Fruc_21_14N_I	RNA	14	AGTGGTCT	DNA	11	TTGGCCTT
KLD3_Fruc_21_14N_I	RNA	15	CTACCAAG	DNA	12	CCTAATGG
KLD3_Fruc_21_14N_I	RNA	16	CTGACTCT	DNA	13	CTGTGAGT

Sample	Template	Fraction	Barcode	Template	Fraction	Barcode
KLD3_Fruc_21_14N_I	RNA	17	AGTGTGAG	DNA	14	GACTGACT
KLD3_Fruc_21_14N_I	RNA	18	GTTCGATG	DNA	15	CTGATGTG

Supplementary Table 2. Confusion matrix for the training data obtained from a Random forest model using all strains and labelling levels.

Actual/predicted	0	5	10	25	50	100	Class. error
0	177	39	17	6	2	2	0.27
5	38	163	17	8	3	0	0.29
10	3	7	202	4	13	0	0.12
25	11	4	15	196	18	1	0.20
50	0	3	7	15	154	11	0.19
100	5	0	0	1	28	207	0.14

Supplementary Table 3. Confusion matrix for classifying *E. coli* test data obtained from a Random forest model using all strains but *E. coli* and all labelling levels.

Actual/ predicted	0	5	10	25	50	100
0	36	1	0	0	0	0
5	27	0	0	0	0	0
10	3	1	1	3	11	1
25	18	4	0	5	2	0
50	0	0	0	0	14	4
100	0	0	0	0	11	20

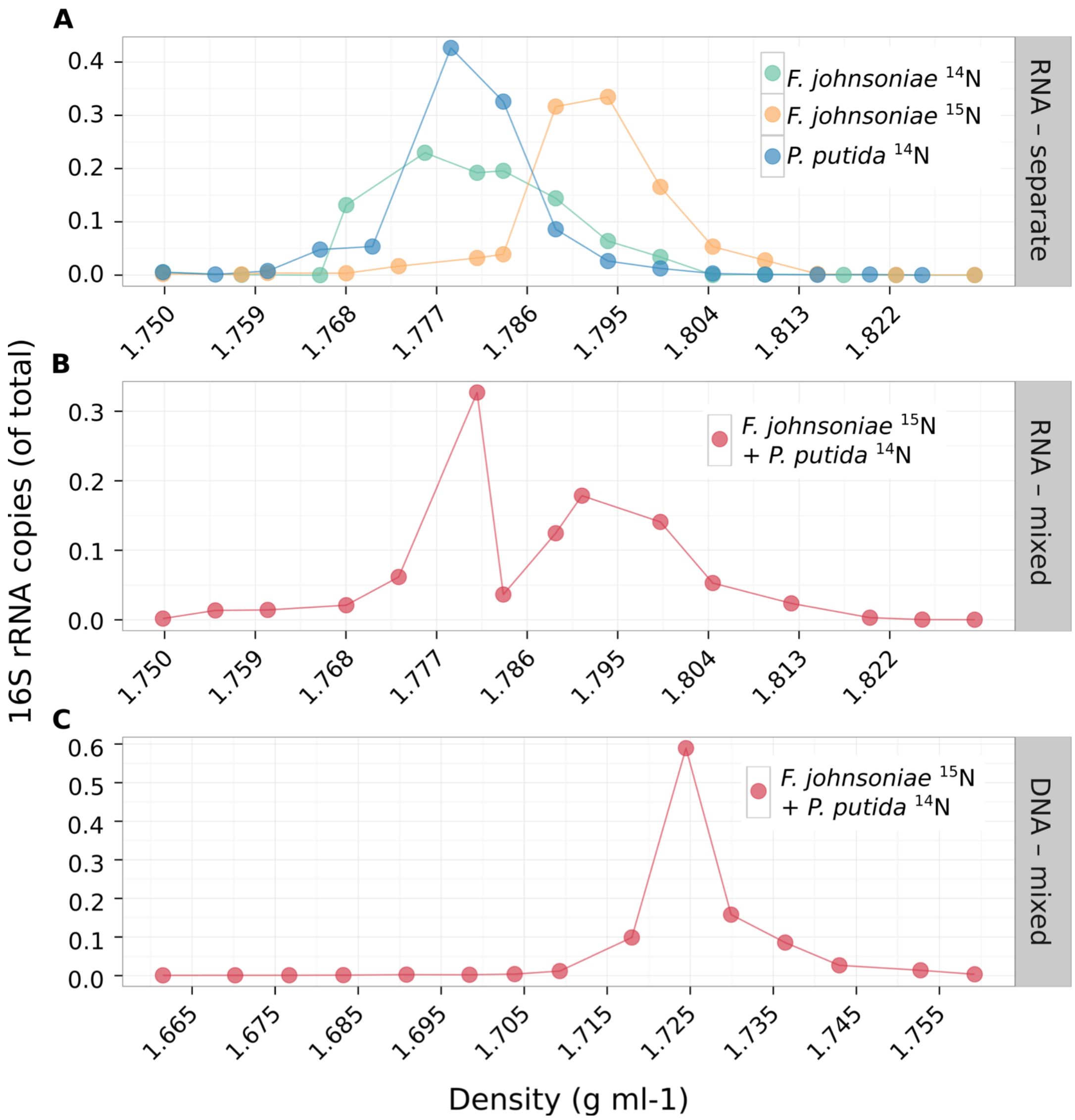

Supplementary Figure 1. Proportion of bacterial 16S rRNA copies recovered from each of the SIP gradient fractions from pure cultures. Values on the Y-axis represent the proportion of the RNA copies out of the total number of RNA copies of the entire gradient. A. RNA-SIP (CsTFA density gradients) of each culture type run independently. B. RNA-SIP of a mixture of the two cultures. C. Primary DNA-SIP (CsCl density gradients) of the two cultures.

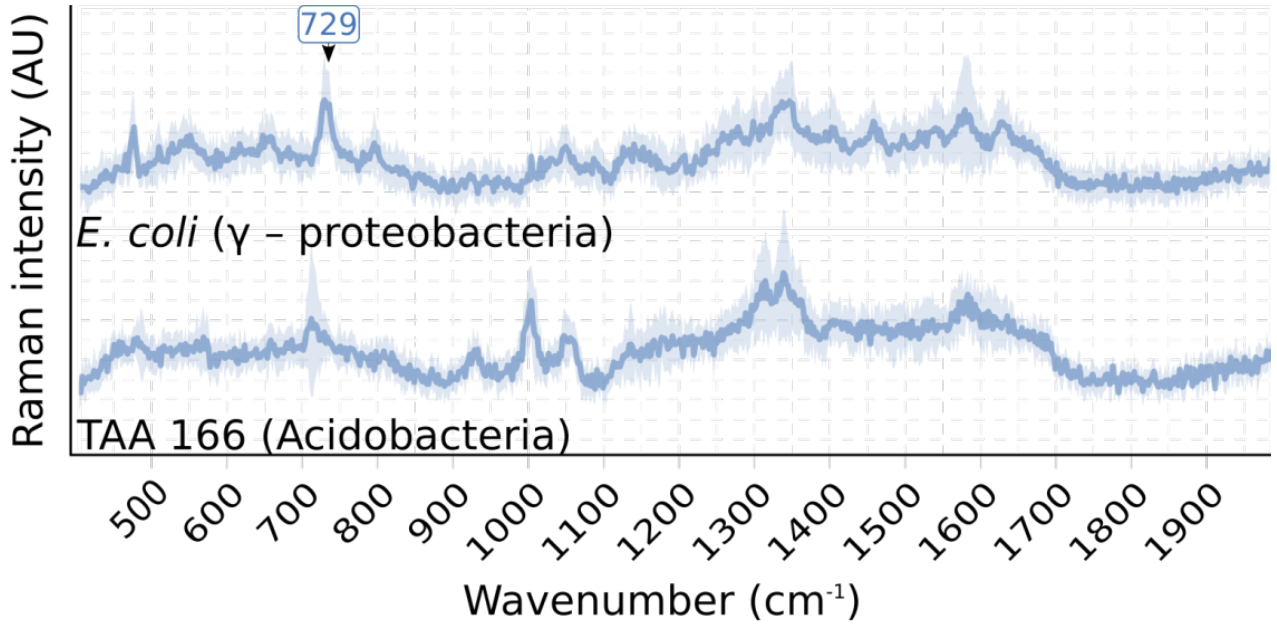

Supplementary Figure 2. SERS spectra of unlabelled *E. coli* and *Acidobacteriaceae* bacterium TAA 166 cells. Means (bold lines) and standard error (light bands) are depicted (n = ~ 10). Number label indicates the position of the peak associated with adenine compounds.
